# Hypercoagulable State and Thrombosis of Bioprosthetic Transcatheter Aortic Valve Replacement (TAVR) Refractory to Common Anticoagulation Methods in the Setting of Protein S Deficiency

**DOI:** 10.7759/cureus.38754

**Published:** 2023-05-09

**Authors:** Caleb J Kellam, Jurxhin Derraj, Gregory M Furletti, Bibai Ren, Toral Patel

**Affiliations:** 1 Medical School, Liberty University College of Osteopathic Medicine, Lynchburg, USA; 2 Internal Medicine, Hospitalist Associates of Virginia, Lynchburg, USA; 3 Heart and Vascular Institute, Centra Health, Lynchburg, USA

**Keywords:** enoxaparin, aortic stenosis, apixaban, venous thromboembolism, warfarin, protein s deficiency, hereditary protein s deficiency, acquired protein s deficiency, bioprosthetic aortic valve thrombosis, tavr

## Abstract

Protein S deficiency is a form of thrombophilia in which the anticoagulant protein S is underproduced or not produced at all by the body. Lifelong anticoagulation is the mainstay of treatment. Transcatheter aortic valve replacement (TAVR) is a current treatment modality for patients with severe aortic stenosis. We are reporting the case of a patient with this disease who underwent a TAVR procedure and experienced valve leaflet thrombosis and large arterial thrombosis in the following months while fully anticoagulated with typical anticoagulation methods including warfarin, apixaban, and enoxaparin. Literature-based guidance is lacking with regard to anticoagulation in the setting of TAVR patients, especially in those with protein S deficiency. Based on our observations, warfarin was the better long-term prophylactic management method for our patient’s protein S deficiency. Enoxaparin was most useful during periods of elevated thrombosis risk, including intra-/post-operative care and prolonged hospitalization periods. In the setting of her TAVR, we observed that warfarin use with a target international normalized ratio (INR) of 2.5-3.5 was the most effective outpatient treatment for the reversal of thrombosed bioprosthetic valve and improvement of cardiac ejection fraction. It is also possible that initial post-operative warfarin use would have been the most effective means of preventing valve thrombosis entirely in our protein S-deficient patient.

## Introduction

Protein S deficiency results in the inability to properly control coagulation. Protein S serves as a cofactor for the protein C which is responsible for inactivating factors Va, VIIa, VIIIa, and Xa of the coagulation cascade. Unopposed activation of these factors leads to thrombophilia and development of venous thromboembolisms [1,2]. The treatment modalities can be lifelong and are aimed at preventing complications. Current anticoagulation therapy options include heparin products like enoxaparin, vitamin K antagonists (VKAs) like warfarin, and direct oral anticoagulants (DOACs) like apixaban. In the past, warfarin has been the first-line treatment for most scenarios needing anticoagulation, but more recent clinical trials have shown DOACs to be safer and equally effective in many cases [2-4]. Warfarin use is now reserved for specific patients, including those with extremely high or low body weights, deep vein thrombosis with proximal clot burden, and submassive or massive pulmonary embolisms with severe clinical presentations [4]. Even though DOACs are becoming more widely used and studied, there is limited understanding of and evidence for the recommended dosages and durations in patients dealing with various forms of thrombophilia like protein S deficiency [1,2].

Warfarin mechanistically works by reducing the synthesis of active clotting factors. It does this by depleting vitamin K stores through the inhibition of the vitamin K epoxide reductase enzyme. Warfarin has been shown to inhibit procoagulant factors II, VII, IX, and X, as well as, anticoagulant factors C and S. This greatly reduces the body's ability to clot, making patients susceptible to serious adverse effects, like uncontrollable hemorrhage [5]. Since each individual has an unpredictable response to warfarin, each patient is required to have close monitoring to assess whether they are within the therapeutic international normalized ratio (INR) range [2,5,6].

DOACs work by directly inhibiting factors of the coagulation cascade. The effective dosages of DOACs are known, and their corresponding effects are predictable. They are becoming more commonly prescribed due to predictable outcomes, minimal bleeding risk, and no need for close monitoring to assess therapeutic range [7]. Apixaban is a more commonly used drug in this class because it is associated with lower risks of gastrointestinal bleeding compared to the other DOACs [2,8]. It is a reversible, direct factor Xa inhibitor that leads to the inhibition of both the intrinsic and extrinsic pathways of coagulation. Although its adverse effects are not as severe as warfarin's, apixaban use does require dose adjustment in the presence of renal impairment and tighter patient adherence due to its shorter half-life [7,8].

Low-molecular-weight heparin (LMWH) products are typically not a primary outpatient treatment modality because they require IV or subcutaneous injection, which is associated with poor patient adherence. Despite this, they are still frequently used in the hospital setting for thrombosis prophylaxis [9,10]. Enoxaparin is the most widely used LMWH due to its wide range of FDA-approved indications and extensive inclusion in hospital formularies [10]. Enoxaparin functions as an indirect anticoagulant, potentiating antithrombin III to irreversibly inactivate factor Xa [9,10]. It has highly predictable outcomes and does not require close monitoring. Before starting therapy, a patient's hepatic function, renal function, and bleeding risk need to be assessed. Bleeding is the most common adverse effect of LMWH. Additionally, dose adjustment is needed in the event of hepatic or renal impairment [9,10]. It is common practice to use LMWH as a bridge to warfarin or DOACs. These products could also be explored as the primary treatment of thrombophilia patients after the failure of other therapies [1,10]. These hypercoagulable patients with more severe diseases, such as protein S deficiency, need closer monitoring when there is a failure of commonly used anticoagulation methods. 

Transcatheter aortic valve replacement (TAVR) is increasingly becoming a more desirable treatment option for critical aortic stenosis. A common concern with this procedure is valve leaflet thrombosis, which will cause reduced valvular motion and ejection fraction [11]. Post-operative anticoagulation for this specific procedure is poorly documented in patients with protein S deficiency [11,12]. The established treatment methods in the general population may not be enough to prevent valvular thrombosis and dysfunction in these patients. In the event of TAVR thrombosis, there is no documentation to guide anticoagulation management in these unfortunately predisposed patients.

## Case presentation

The patient is an 80-year-old Caucasian female with a history of lifelong tobacco use, long-term anticoagulation with warfarin, rheumatoid arthritis, chronic steroid use with immunosuppression, gastric bypass, hysterectomy, osteoporosis, hypertension, and recurrent deep vein thrombosis and pulmonary embolism secondary to protein S deficiency. Her protein S deficiency diagnosis was reached during a hematological workup after multiple thromboembolic events. Twenty-three years ago (1999), she had an unprovoked right lower extremity deep vein thrombosis requiring urokinase, thrombectomy, and four-compartment fasciotomy. Eighteen years ago (2004), she had a pulmonary embolism while recovering from gastric bypass surgery. An inferior vena cava (IVC) filter was placed as a prophylaxis for recurrent pulmonary emboli and the patient was placed on 1 mg warfarin daily with a target INR of 2-3, on which she has had no thromboembolic events for the last 18 years.

**Figure 1:**
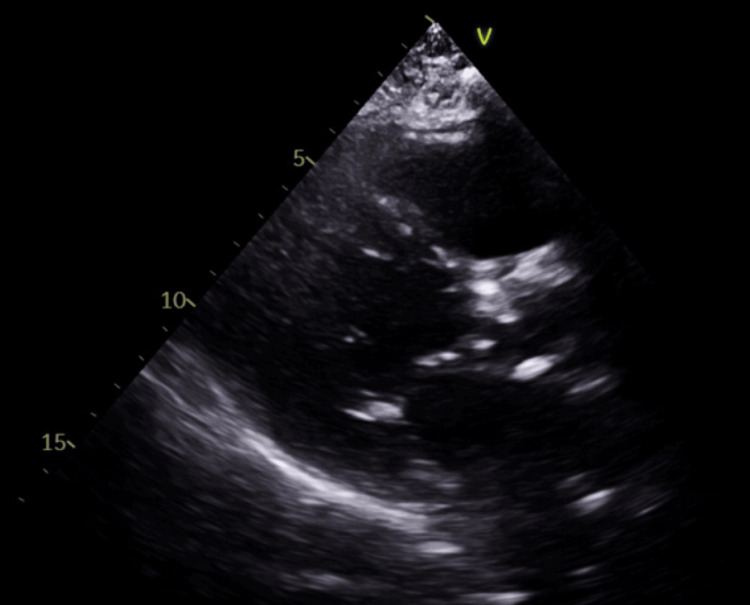
Pre-TAVR Stenosed Aortic Valve Ultrasound parasternal long-axis view: severely calcified aortic stenosis TAVR: transcatheter aortic valve replacement

Our patient first presented to the emergency department with chest pain and gastrointestinal bleeding. She was transfused with one unit of packed red blood cells and her warfarin was discontinued without reversal. She was diagnosed with non-ST-elevation myocardial infarction (NSTEMI), which was confirmed with serial serum troponin-I peaking at 16.96 ng/dL. A heart catheterization revealed mild coronary artery disease without the need for revascularization. An echocardiogram revealed severe aortic stenosis assumed to be of non-rheumatic origin (Figure 1). Heavily calcified aortic valve leaflets and mild iliofemoral vessel atherosclerotic disease were also observed. She was not a candidate for open aortic valve replacement and was placed on a low dose of 2.5 mg apixaban twice daily instead of reinstituting her warfarin therapy. She was discharged three days later with plans for TAVR within one to two weeks.

**Figure 2:**
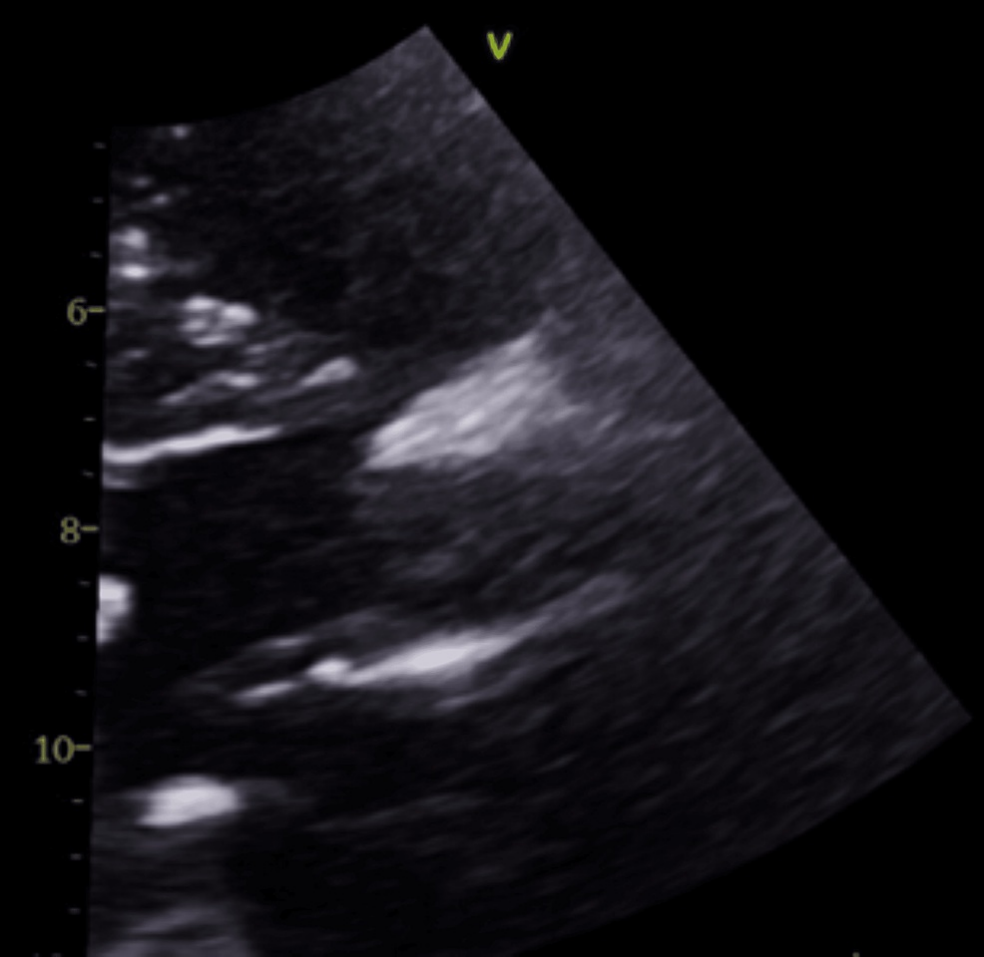
Post-TAVR Parasternal Long-Axis View Image taken one day following procedure. TAVR: transcatheter aortic valve replacement

**Figure 3:**
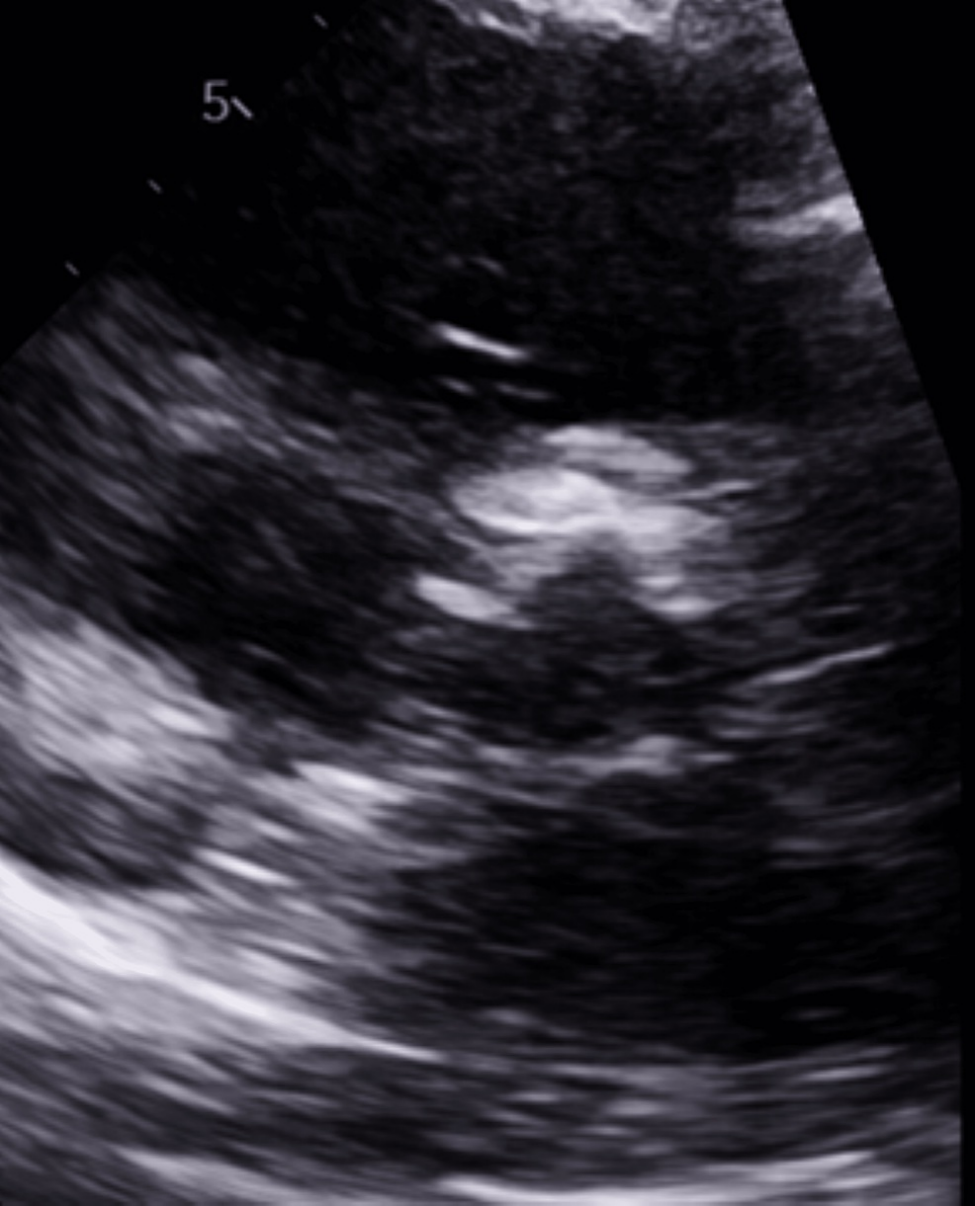
Post-TAVR Parasternal Short-Axis View Image taken one day following procedure TAVR: transcatheter aortic valve replacement

Ten days after discharge, she once again presented to the emergency department with a recurrence of chest pain and elevated troponins peaking at 1.72 ng/dL. Two days later, she underwent the TAVR procedure prior to her scheduled surgery date, during which an Edwards SAPIEN bioprosthetic bovine pericardium valve (Edwards Lifesciences, Irvine, California, United States) was used. Vascular access was obtained through the left common femoral vein and right common femoral artery. Her apixaban was stopped 48 hours pre-operatively. The procedure was uneventful. She resumed apixaban 24 hours post-operatively without bridging. A transthoracic echocardiogram (TTE) confirmed the correct placement of the bioprosthetic valve immediately following the procedure (Figures 2, 3). The following day, another TTE was performed, measuring an ejection fraction (EF) of 60-65% with normal wall motion, a mean systolic gradient of 18 mmHg with a peak systolic gradient of 28 mmHg, left ventricular outflow tract (LVOT) to aortic valve (AV) mean velocity ratio of 0.3, and an LVOT to AV velocity time integral (VTI) ratio of 0.38. At this time, there were no signs of stenosis or regurgitation across the newly placed valve.

**Figure 4:**
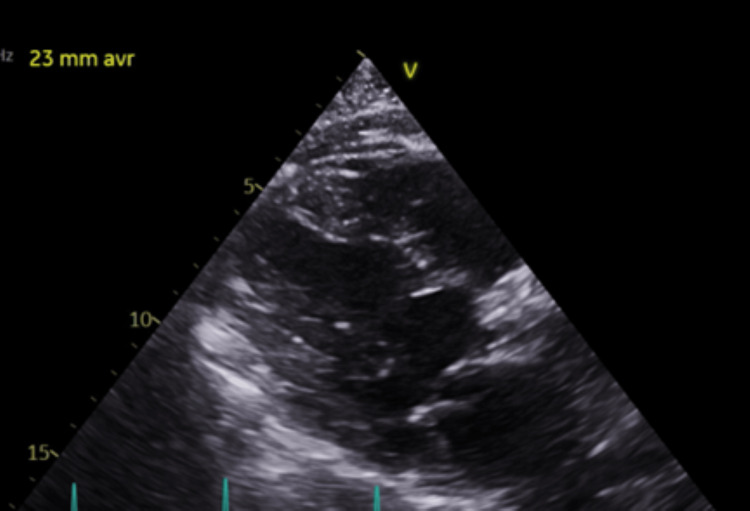
Six Weeks Post TAVR. Thrombosed Valve while on Apixaban Ultrasound parasternal long-axis view showing thickening of bioprosthetic valve leaflets. TAVR: transcatheter aortic valve replacement

**Figure 5:**
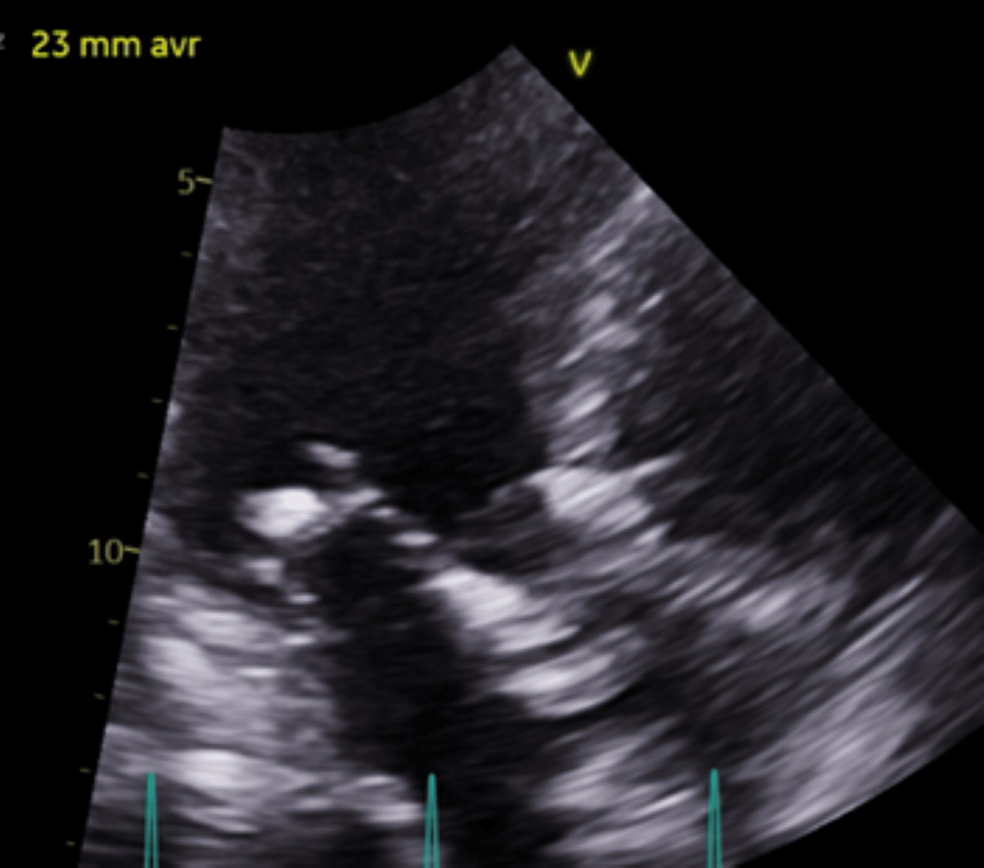
Six Weeks Post TAVR. Thrombosed Valve while on Apixaban Ultrasound three-chamber long-axis view showing thickening of bioprosthetic valve leaflets. TAVR: transcatheter aortic valve replacement

**Figure 6:**
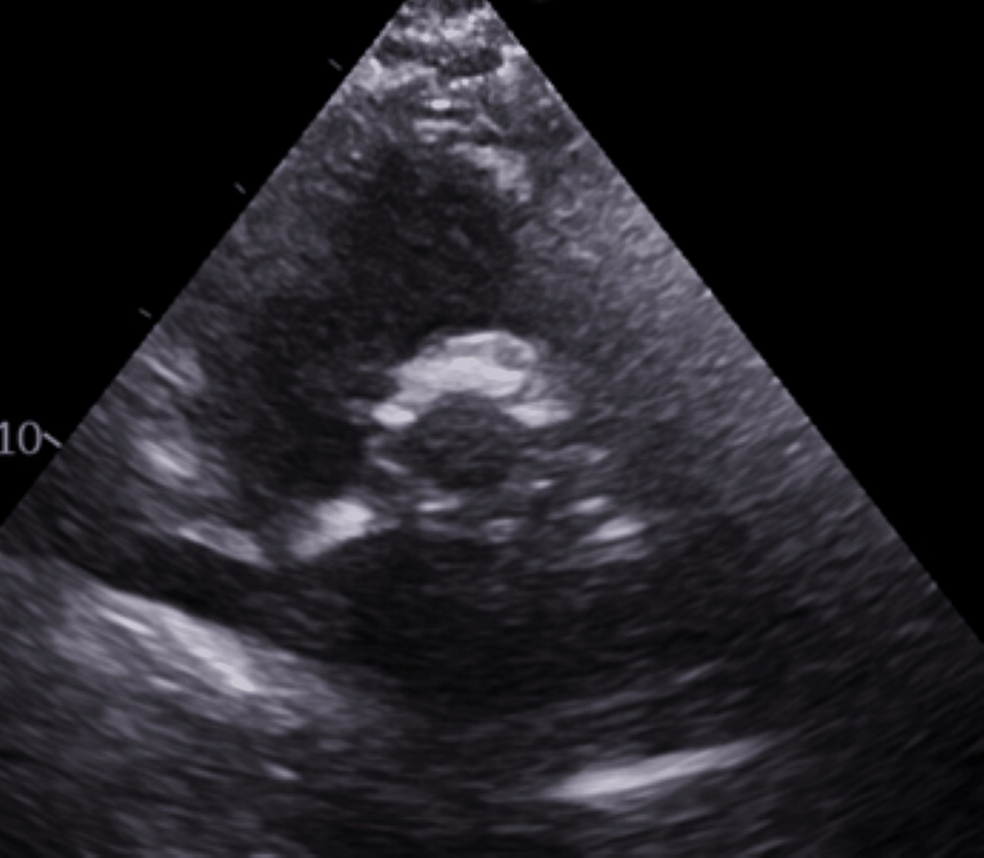
Six Weeks Post TAVR. Thrombosed Valve while on Apixaban Ultrasound parasternal short-axis view showing thickening of bioprosthetic valve leaflets. TAVR: transcatheter aortic valve replacement

After seven weeks post-operation, she underwent an outpatient TAVR follow-up TTE which revealed an increased valve velocity with an EF of 55-65% without regurgitation (Figures 4-6). The mean systolic gradient increased from her previous measurement to 37 mmHg with a peak systolic gradient of 69 mmHg. The noted changes in these metrics raised suspicions of bioprosthetic valve leaflet thrombosis in the setting of her hypercoagulable state secondary to protein S deficiency. Her apixaban was increased to 5 mg twice daily with a repeat TTE scheduled for three weeks later.

**Figure 7:**
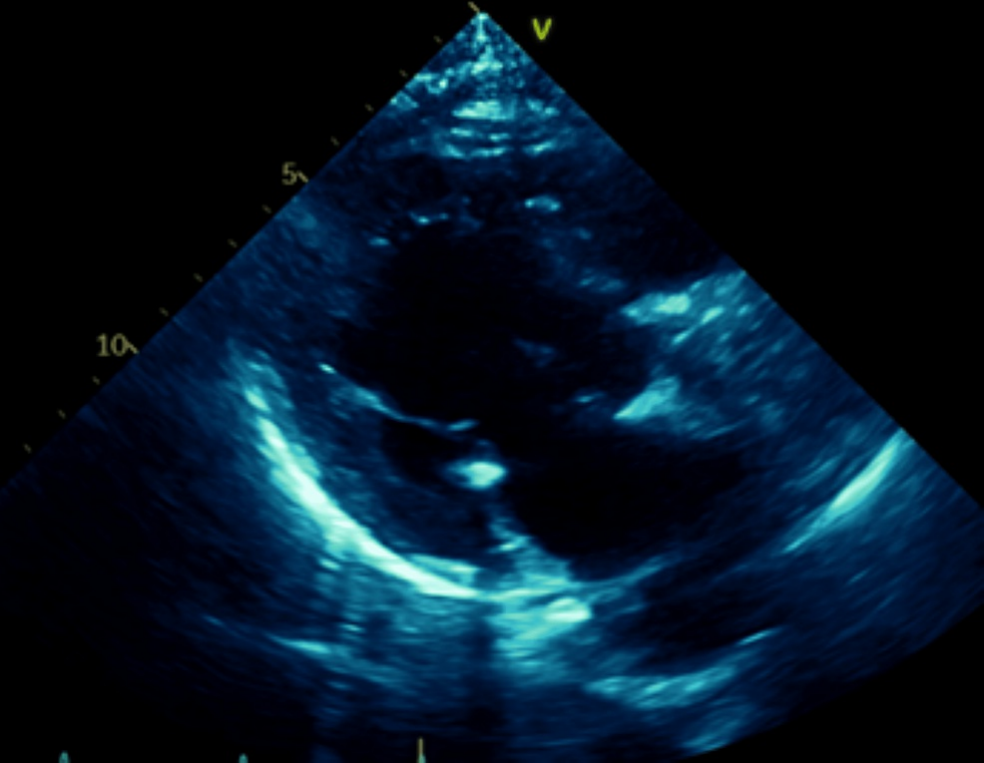
Eight Weeks Post TAVR. Thrombosed Valve while on Apixaban Ultrasound parasternal long-axis view showing thickening of bioprosthetic valve leaflets. TAVR: transcatheter aortic valve replacement

**Figure 8:**
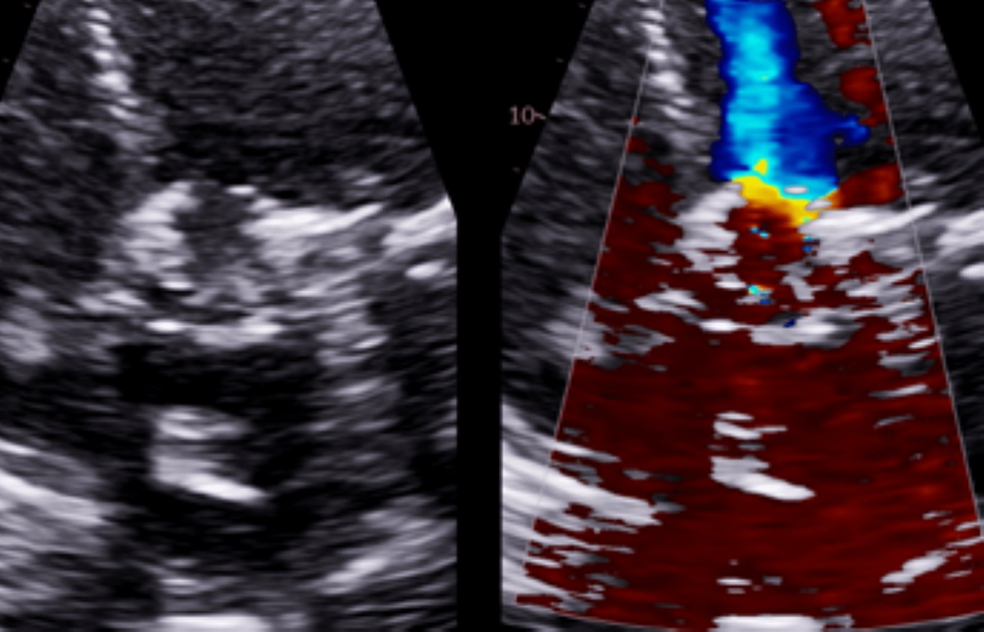
Eight Weeks Post TAVR. Thrombosed Valve while on Apixaban Ultrasound five-chamber long-axis view with color doppler showing flow acceleration across the bioprosthetic aortic valve. TAVR: transcatheter aortic valve replacement

**Figure 9:**
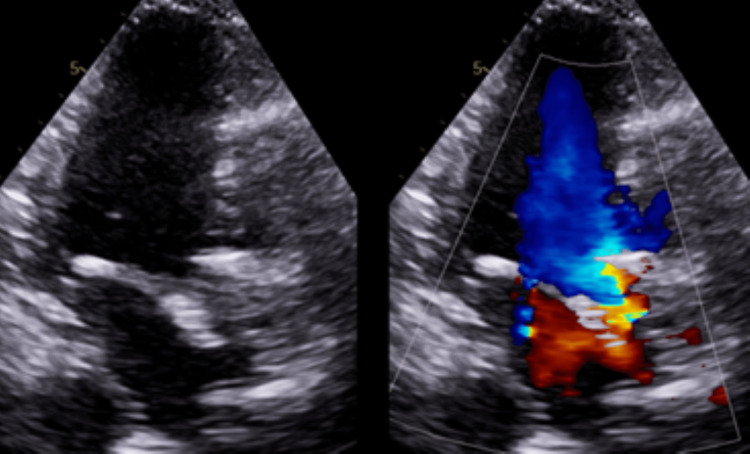
Eight Weeks Post TAVR. Thrombosed Valve while on Apixaban Ultrasound three-chamber long-axis view with color doppler showing flow acceleration across the bioprosthetic aortic valve. TAVR: transcatheter aortic valve replacement

**Figure 10:**
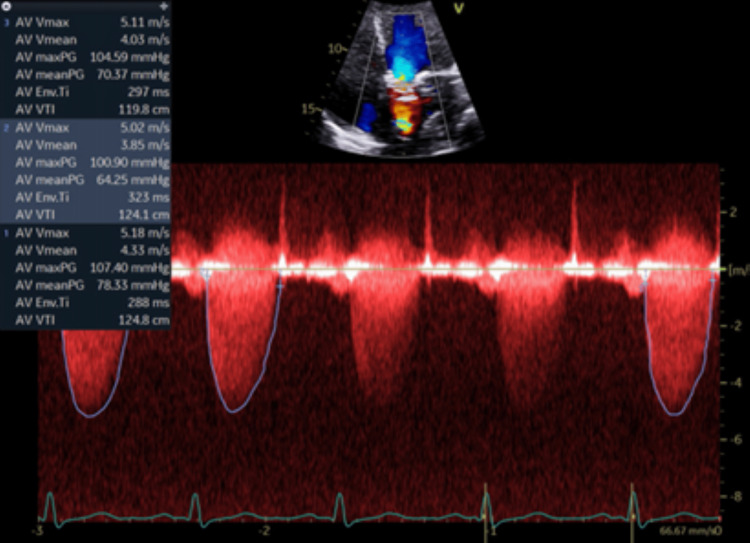
Eight Weeks Post TAVR. Thrombosed Valve while on Apixaban Color-flow doppler and velocity mapping of aortic valve showing increased transvalvular velocity with peak velocity 5.18m/s, mean gradient 78mmHg, and peak gradient 107mmHg. TAVR: transcatheter aortic valve replacement; AV: aortic valve

Two weeks later, the patient presented to the emergency department a third time for chest pain. A TTE was immediately ordered, which showed a further decrease in EF to 40-45% with diffuse, mild hypokinesis (Figures 7-9). There were dramatic increases in aortic valve peak systolic velocity and mean systolic gradient to 5.2 m/sec and 78 mmHg, respectively, with a peak systolic gradient of 107 mmHg (Figure 10). The LVOT to AV VTI ratio was 0.34. Troponin and ECG studies were unremarkable. A trans-esophageal echocardiogram (TEE) revealed thickening of all bioprosthetic aortic valve leaflets with severe stenosis of the valve. The leaflet separation was reduced with a valve area of 0.4 cm^2^ and no evidence of vegetation. Upon hospitalization, apixaban was discontinued and enoxaparin 65 mg was administered every 12 hours due to the possible need for surgical intervention. The patient was not a procedural candidate at this time, so the decision was made to treat the thrombosed valve medicinally. Her valve thrombosed despite apixaban therapy, so prior to discharge she was placed back on warfarin at a dose of 2 mg daily with a target INR of 3 within the range of 2.5-3.5.

Approximately 17 days after this visit, the patient presented to the emergency department a fourth time with acute chest pain and shortness of breath. Chest X-ray revealed bilateral basilar lung opacities on chest x-ray. Laboratory analysis revealed a serum BNP of 2,838 pg/mL. A promptly obtained TTE revealed a further reduction in EF to 25-30%. The aortic valvular peak velocity improved to 4.2 m/s and the mean systolic gradient was improved at 50 mmHg, with a peak gradient of 71 mmHg. She was diagnosed with acute heart failure with reduced EF (HFrEF) secondary to her bioprosthetic aortic valve thrombosis. She was placed on 20 mg daily of furosemide indefinitely. Her warfarin therapy was continued.

One week after discharge, the patient attended an outpatient chronic heart failure (CHF) follow-up. A TTE was obtained that revealed an EF of 25-30%, stroke-volume index of 39, LVOT to AV VTI ratio of 0.34. She continued with 20 mg of furosemide daily. Two weeks after this, she underwent outpatient cardiology follow-up. A TTE was obtained and revealed an improved EF of 35-40%.

Twenty-three days later, the patient presented to the emergency department with pain in her right leg, which was cold and without distal pulses upon palpation. She was admitted with Rutherford’s acute ischemia of the right lower extremity secondary to right-sided common and external iliac artery thrombosis. Her INR was supratherapeutic at 4.2. She underwent emergent vascular surgery with right femoral artery cutdown, catheter placement in superficial femoral artery, and open thrombectomy of the right superficial and common femoral, popliteal, and common and external iliac arteries. Warfarin was discontinued and she was indefinitely placed on a 100 mg daily subcutaneous injection of enoxaparin at the agreed-upon request of hematology and vascular surgery specialists. This decision was made due to the severity of her hypercoagulable state and the frequency of thrombotic events. The timeline is given in the Appendix.

## Discussion

Anticoagulating patients with hypercoagulable states secondary to protein S deficiency has not been well documented in current literature. This is likely due to the fact that, in its familial form, its prevalence is estimated to be only 0.03-0.13% of the population. When you focus the data to include only those with a personal history or family history of chronic thromboembolism, the prevalence of protein S deficiency increases to 3-5%. Since the identification of protein S deficiency, the empirical treatment of patients has largely remained undifferentiated from the standard anticoagulation protocols used in the treatment of acute deep venous thromboses (DVTs), acute pulmonary embolisms (PEs), and prevention of emboli in the setting of atrial fibrillation. The main difference in patients like ours is that their anticoagulation therapy will always be lifelong. Almost all symptomatic patients with protein S deficiency present with recurrent, unprovoked venous thromboembolisms (VTEs). In our patient, this has been seen over the course of the last 23 years. It is highly probable that many protein S deficiency patients were treated empirically for VTEs with anticoagulation and their diagnosis of protein S deficiency did not come until recurrence was witnessed [1].

Prior to 2010, outpatient oral anticoagulation was predominantly performed using VKA warfarin. One of its most common indications and uses was in atrial fibrillation patients. Various clinical trials have successfully shown that DOACs have equal efficacy with the added benefit of decreased risk of bleeding and easier outpatient management. From 2011 to 2016, the use of DOACs increased from 21.8% to 76.2% and the use of warfarin decreased from 78.2% to 23.8%. At this time, DOACs are recommended instead of warfarin therapy for patients with atrial fibrillation, but there are no specific and widely accepted guidelines for the anticoagulation of patients with protein S deficiency, especially in the setting of TAVR [13].

Our patient received her protein S deficiency diagnosis when the standard of care for any hypercoagulable state was lifelong warfarin therapy. She was on warfarin with an INR range of 2-3 for 18 years. During this span, she experienced no recurrence of venous thromboembolism, suggesting that this medication was effective in managing her condition.

Upon presenting to our hospital with severe aortic stenosis, it was determined that she needed to undergo a TAVR procedure. During the same hospital stay, she was also treated for a gastrointestinal bleed. Following the precedent established by many of these aforementioned clinical trials, her warfarin was discontinued in favor of apixaban due to its association with decreased risk for further bleeding.

Our patient underwent a successful TAVR procedure without complication. Approximately 50 days later, despite the ongoing apixaban therapy, a TTE revealed decreased EF due to thrombosis of the bioprosthetic aortic valve leaflets. Initially, this was thought to be because a higher dose of apixaban was required, leading to the decision to double the dose. However, two weeks later, she was readmitted with a worsened EF with symptoms of severe aortic stenosis. Our patient was not a candidate for surgical intervention and a second TAVR procedure would risk shedding emboli from the previously placed thrombosed valve. It was a reasonable decision to place her back on the warfarin therapy that she had been on for the last two decades. In the setting of her new bioprosthetic valve, her INR goal was 2.5-3.5. Approximately 18 days later, when hospitalized for HFrEF, her EF had declined to 25-30%. Her mean systolic gradient improved from 78 mmHg to 50 mmHg, with her peak systolic gradient changing from 107 mmHg to 71 mmHg. In addition to this, it did not appear as if her valve had thrombosed further, indicating that her warfarin therapy was effective. Her overall condition improved with diuresis alone. Three weeks later, an outpatient TTE revealed further improvements in aortic valve functioning. This time her EF had improved to 35-40%, indicating that the warfarin therapy continued to be effective.

Unfortunately, one week later, our patient developed right common and external iliac artery thrombosis. Of note, this is proximal to the right superficial femoral artery, the same artery which was used to obtain vascular access during her TAVR procedure exactly 111 days prior. Interestingly, her INR at the time was at a supratherapeutic level of 4.2. In other words, she was susceptible to severe hemorrhage while simultaneously experiencing critical thrombosis of a large artery. After her emergent vascular surgery, it was deemed too dangerous to place her back on warfarin. Even in the outpatient setting, she will have to remain anticoagulated with enoxaparin for the foreseeable future.

This case provides valuable insight into both the general management of patients with protein S deficiency and the specific management of these patients after a TAVR procedure. Based on observations of this case, warfarin therapy could potentially be the best treatment option for patients with protein S deficiency in the unprovoked setting. Anecdotally, our patient experienced 18 years of remission from thromboembolisms due to warfarin therapy. Based on our physiological understanding of warfarin, it is likely more effective than DOACs in the setting of protein S deficiency because it removes the homeostatic need for protein S in the body by inhibiting key extrinsic and intrinsic coagulation cascade factors, including protein C and S themselves. Although it may not be the most widely accepted treatment, it is clear in patients similar to ours in the setting of TAVR placement, warfarin should be considered as a primary modality of outpatient anticoagulation. This is supported by both the rapidly observed thrombosis and decrease in function of her new valve while on apixaban therapy, and her gradual improvement in valve functioning once switching back to warfarin. It is possible an earlier switch back to warfarin could have prevented her valve's thrombosis entirely.

Now we must address the additional problem of how, even while her valve function was improving, she managed to develop a substantial arterial thrombus. Of note, this same right lower extremity underwent open thrombectomy and fasciotomy 23 years ago for DVT. It was also the source of vascular access during her TAVR procedure after it had already been noted on CT angiogram that there was mild iliofemoral atherosclerotic disease. It is not only possible but highly probable that this predisposed the patient to thrombus in the setting of her protein S deficiency and ineffective apixaban therapy. We are unsure that an early switch back to warfarin would have prevented this outcome for her with regard to her arterial thrombus; however, the benefits of warfarin on her thrombosed bioprosthetic valve are clear.

We hypothesize that warfarin could potentially be the better empiric therapy for all healthy non-hospitalized patients with protein S deficiency, but that in the setting of older age, recurrent hospitalizations, and mounting cardiovascular comorbidities, enoxaparin would be best because these patients exist in a hypercoagulable state. Additional research is clearly needed to substantiate our hypotheses and make sense of our observations. The question “what should be the empiric therapy for protein S deficiency?” needs more extensive documentation. Furthermore, the question “what anticoagulant should be used in TAVR patients with protein S deficiency?” also needs to be addressed.

## Conclusions

Patients with protein S deficiency require special consideration when being placed on anticoagulation, both in the setting of standard venous thromboembolism prophylaxis and in the prevention of bioprosthetic valve thrombosis. Based on what we witnessed, the better course of action for protein S-deficient patients undergoing TAVR procedures could be warfarin therapy instead apixaban therapy. When these patients present with additional risk factors and comorbidities, chronic treatment with enoxaparin could be considered, as even warfarin therapy may not be sufficient to manage them in these scenarios.

## Appendices

**Table 1:** Sequence of Events with Corresponding Changes in Anticoagulation Method Tracking the patient's timeline of emergency department (ED) visits, hospital admissions, and follow-up appointments. TAVR: transcatheter aortic valve replacement; NSTEMI: non-ST-elevation myocardial infarction

Timeline:	Description:	Diagnosis:	Changes in Anticoagulation Therapy	Ejection Fraction:
1st Admission	Initial ED Visit	NSTEMI Secondary to Severe Aortic Stenosis	Warfarin 1.0mg to Apixaban 2.5mg	
+15 days	Post-TAVR Placement		No Changes	60-65%
+50 days	Cardiology Follow-Up	Valve Thrombosis	Apixaban 2.5mg to 5.0mg	55-65%
+13 days	ED Visit/Admitted	Valve Thrombosis	Apixaban 5.0mg to Warfarin 2.0mg	40-45%
+17 days	ED Visit/Admitted	Heart Failure Secondary to Valve Thrombosis	No Changes	25-30%
+7 days	Cardiology Follow-Up		No Changes	35-40%
+23 days	ED Visit/Admitted	Arterial Thrombus of Right Lower Extremity	Warfarin 2.0mg to Enoxaparin 100mg	
